# A study of innate immune kinetics reveals a role for a chloride transporter in a virulent *Francisella tularensis* type B strain

**DOI:** 10.1002/mbo3.1170

**Published:** 2021-03-24

**Authors:** Lisa M. Matz, Joseph F. Petrosino

**Affiliations:** ^1^ The Alkek Center for Metagenomics and Microbiome Research Baylor College of Medicine Houston TX USA; ^2^ The Department of Molecular Virology and Microbiology Baylor College of Medicine Houston TX USA; ^3^ Baylor College of Medicine Houston TX USA

**Keywords:** *Francisella tularensis* subsp. *holarctica*, innate immunity, Live Vaccine Strain, proton:chloride antiporter, TargeTron™ chromosomal insertion, tularemia

## Abstract

Tularemia is a zoonotic disease of global proportions. *Francisella tularensis* subspecies *tularensis* (type A) and *holarctica* (type B) cause disease in healthy humans, with type A infections resulting in higher mortality. Repeated passage of a type B strain in the mid‐20th century generated the Live Vaccine Strain (LVS). LVS remains unlicensed, does not protect against high inhalational doses of type A, and its exact mechanisms of attenuation are poorly understood. Recent data suggest that live attenuated vaccines derived from type B may cross‐protect against type A. However, there is a dearth of knowledge regarding virulent type B pathogenesis and its capacity to stimulate the host's innate immune response. We therefore sought to increase our understanding of virulent type B in vitro characteristics using strain OR96‐0246 as a model. Adding to our knowledge of innate immune kinetics in macrophages following infection with virulent type B, we observed robust replication of strain OR96‐0246 in murine and human macrophages, reduced expression of pro‐inflammatory cytokine genes from “wild type” type B‐infected macrophages compared to LVS, and delayed macrophage cell death suggesting that virulent type B may suppress macrophage activation. One disruption in LVS is in the gene encoding the chloride transporter ClcA. We investigated the role of ClcA in macrophage infection and observed a replication delay in a *clcA* mutant. Here, we propose its role in acid tolerance. A greater understanding of LVS attenuation may reveal new mechanisms of pathogenesis and inform strategies toward the development of an improved vaccine against tularemia.

## Graphical abstract

A better understanding of how Francisella tularensis type B strains behave in macrophages is required to further elucidate the attenuating mechanisms of the Live Vaccine Strain, which is not FDA approved. Here we evaluate innate immune kinetics in human and murine macrophages following infection with a virulent type B strain compared with its attenuated counterpart, LVS, and a clcA mutant. Our results suggest that the disruption of the chloride transporter ClcA is one contributor to LVS attenuation and may be a target for a live attenuated vaccine against tularemia.

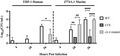

## 1 INTRODUCTION

*Francisella tularensis* (*F. tularensis*) is an intracellular, Gram‐negative coccobacillus and the causative agent of the zoonotic disease tularemia. Two clinically relevant subspecies in humans are subspecies *tularensis* (type A) and *holarctica* (type B), with type A(.I) infections resulting in higher mortality (14%) compared to type B (7%; Kugeler et al., 2009; Staples et al., 2006). *F. tularensis* is classified as a Tier One select agent due to its low infectious dose, high morbidity via the inhalational route, and lack of a licensed vaccine (Centers for Disease Control & Prevention; Kaufmann et al., 1997). During a medical exchange mission with the USSR in 1956, the United States acquired a Live Vaccine Strain, LVS (Public health monograph No. 50. United States‐U.S.S.R medical exchange missions, 1956. Public Health Reports [PHS No. 536]). LVS dramatically decreased laboratory‐acquired tularemia incidence rates, replacing the heat‐killed Foshay vaccine. However, LVS does not protect against high doses of inhalational type A in animals and humans and is thus considered only partially effective (Burke, 1977; Eigelsbach & Downs, 1961; Sawyer et al., 1966). Additionally, LVS is difficult and expensive to manufacture owing to non‐immunogenic colony variants that emerge during its production. LVS remains unlicensed after seven decades of research and development, and the characterization of its attenuating mechanisms is incomplete.

Despite these obstacles, LVS is frequently used as a model organism to study the pathogenesis of virulent type A strains, as their genomes possess more than 97% sequence similarity. However, dozens of rearrangements occur in type A compared to type B genomes, confounding direct comparisons of LVS to type A strains. Differences between type A and type B likely stem from insertional elements and regulatory sequences, in addition to (pseudo)gene content and genome organization. In contrast, differences between LVS and virulent type B strains mainly occur within coding sequences (Petrosino et al., 2006). Although the exact progenitor for LVS is unknown, virulent or “wild type” (WT) type B strains are the closest genetic progenitor of LVS and are therefore the most appropriate context for understanding attenuating mechanisms in LVS.

An important yet often overlooked feature distinguishing virulent type B strains from LVS is that WT type B has a case fatality rate of approximately 7%, whereas, no deaths from LVS have been reported (Staples et al., 2006). While extensive epidemiological and phylogeographical data exist for virulent type B strains, they are understudied compared to type A in terms of pathogenesis and the host's innate immune response. Only a handful of studies have measured WT type B replication and infection kinetics within macrophages or other antigen‐presenting cells (APCs). Ray et al. demonstrated the ability of strain OR96‐0246 to replicate in bone marrow‐derived macrophages (BMDMs) from Fischer 344 rats and reported that its replication in rat hepatocytes was as robust as type A strain Schu S4 (Ray et al., 2010). The growth of strain FSC200 in murine BMDMs, bone marrow‐derived dendritic cells (BMDCs), and in the monocyte cell line J774.2 has also been reported, with both bacterial and host proteomic analyses performed (Bauler *et al*., 2014; Fabrik et al., 2018; Pávková et al., 2013; Straskova et al., 2012). Lindgren et al. explored differences in iron content between several type A and type B strains and correlated these differences to increased susceptibility of type B strains to H_2_O_2_‐mediated killing (Lindgren et al., 2011). Brown et al. monitored serum from North American cottontail rabbits infected with type B strains OR96‐0246 and KY99‐3387 and reported a strong humoral response in rabbits that survived the past 14 days (Brown et al., 2015). Many studies of virulent type B strains typically focus on clinical or diagnostic aspects of tularemia but lack mechanistic insights (Adcock et al., 2014; Fritzsch & Splettstoesser, 2010; Johansson et al., 2000, 2014; Stenmark et al., 2003; Versage et al., 2003).

Using the targeted approach of comparative genomics, our lab and others previously aligned the LVS genome to those of WT type B strains and identified 17 genes that are disrupted in LVS but remain intact in WT strains (Petrosino et al., 2006; Rohmer et al., 2006). This list includes proteins involved in ion transport, sugar modification, protein secretion, nutrient acquisition, and intracellular survival within macrophages, along with genes of unknown function. Only two genes identified in the above studies were explored more extensively. The first encodes a chimeric protein formed by the fusion of two neighboring siderophore genes, *fup* *A*, and *fup* *B* (FTL_RS02265). The type A *fupA* mutant showed reduced virulence in an intradermal mouse infection model; however, the LD_50_ was 10‐fold lower than LVS, suggesting LVS is attenuated by additional mechanisms (Twine et al., 2005). The second gene encodes a type IV pilus assembly protein, PilA (FTH_RS02055). It was separately shown that *pilA* deletions in type A and type B strains are attenuated in mice by the subcutaneous route, but only slightly impaired for intracellular replication in vitro (Forslund et al., 2010; Salomonsson et al., 2009). Salomonsson et al. concluded that since the reintroduction of both *pilA* and *fupA* together restores virulence of LVS in C57BL/6 mice to a level similar to that of WT type B strains, these genes are therefore responsible for LVS attenuation. However, this finding does not preclude the possibility that the reintroduction of other candidate genes could equally restore virulence in LVS. Furthermore, limitations exist in C57BL/6 mice compared to other animal models, as immunization with LVS generally does not protect C57BL/6 mice against type A challenge (Griffin et al., 2014). While the above findings indicate an important role for FupA and PilA, the contribution of the remaining individual gene disruptions to LVS attenuation has yet to be determined, and their roles in virulence are unclear.

**FIGURE A1:**
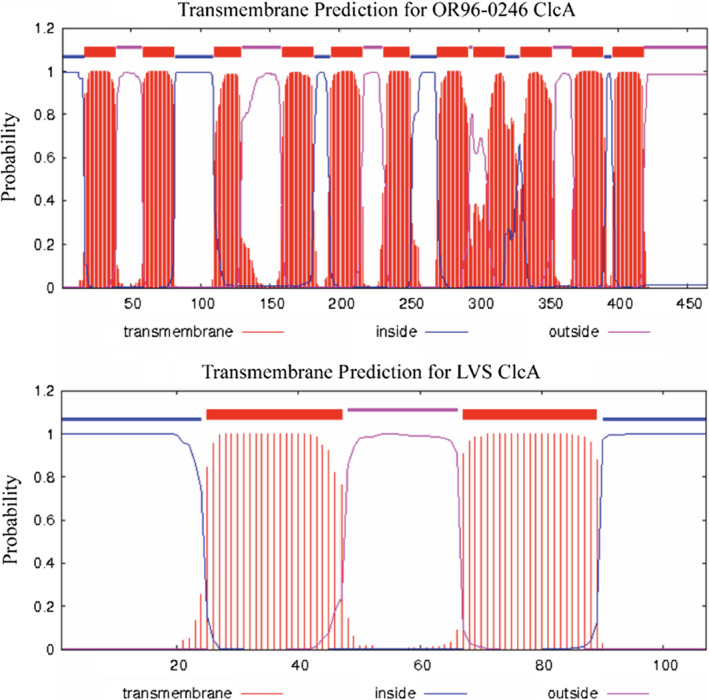
Transmembrane prediction of ClcA. Probability of ClcA membrane insertion determined by TMHMM Server v. 2.0 (www.cbs.dtu.dk) for OR96‐0246 (top, AAX59_00075) and LVS (bottom, FTL_0102). Amino acid length is displayed on the x‐axis and probability on the y‐axis. Red regions represent helices within ClcA predicted to be inserted into the membrane

We have retargeted the parent *Francisella* TargeTron™ plasmid for the disruption of all 17 attenuation candidate genes, laying the groundwork for others to study these genes of interest. One such gene is *clcA*, which encodes a predicted proton:chloride exchange transporter with eleven predicted trans‐membrane spanning regions (Figure A1). Based on studies in the *Escherichia coli* (*E. coli*) homolog, Rohmer et al. suggested that ClcA might be important for survival at low pH (Rohmer et al., 2006). We test this hypothesis here and describe the innate immune response kinetics in human and murine macrophages following infection with a virulent type B strain compared to its attenuated counterpart, LVS, and disruption mutant *clcA*::*ltrB*_Ll_. This study adds to a growing body of work focused on increasing our understanding of virulent type B strains, and to our knowledge is the first‐ever characterization of a *Francisella* chloride channel protein.

## 2 MATERIALS AND METHODS

### 2.1 Bacterial strains and stock preparation

A list of strains and plasmids used in this study can be found in Table 1. *F. tularensis* subsp. *holarctica* strain OR96‐0246 NR‐648, originally isolated in Oregon in 1996 after a primate facility outbreak, was obtained through BEI Resources, NIAID, NIH and sequenced at Baylor College of Medicine as previously described (Atkins et al., 2015). *F. tularensis* subsp. *holarctica* LVS was obtained through Dynport Vaccine Company LLC (derived from NDBR101 Lot 4; Pasetti et al., 2008). LVS can be purchased through BEI Resources. Strains were grown at 37°C in modified Mueller‐Hinton (MHII, cation‐adjusted) broth (BD) supplemented with sterile 0.1% glucose, 0.025% ferric pyrophosphate, and 2% reconstituted, sterile IsoVitalex (BD Difco). Bacterial counts were determined by spot plating serial dilutions on MHII agar +5% fetal bovine serum (FBS) and growing at 37°C with 5% CO_2_ for 48 h. Any samples removed from BSL‐3 for further analysis were inactivated and confirmed for sterility by plating at 37°C with 5% CO_2_ for more than 48 h.

**TABLE 1:** Bacterial strains and plasmids used in this study

Strain or Plasmid	Description	References
*Strains*		
OR96‐0246	*Ft* subsp. *holarctica*	ATCC
*clcA* mutant (LMM1)	OR96‐0246 *clcA*::*ltrB*_Ll_	This study
Live Vaccine Strain	*Ft* subsp. *holarctica*	DynPort Vaccine
MG1655	*E. coli* Wild‐type	
MG1655Δ*clcA*Δ*clcB*	*E. coli* CLC double knockout	C. Miller (Iyer et al., 2002)
*Plasmids*		
pFTClcA	IPTG‐inducible plasmid expressing *Ft* ClcA	This study
pKEK1140	*Francisella*‐adapted TargeTron™ vector, Kan^R^	Karl Klose (Rodriguez et al., 2008, 2009)
pJFP1004	pKEK1140 retargeted for *clcA* (FTH_RS00495)	This study
pEZClcA	Synthetic DNA construct encoding *Ft* ClcA, Kan^R^	Epoch Life Sciences
pWSK29	Low‐copy IPTG‐inducible plasmid, Amp^R^	S. Kushner (Wang & Kushner, 1991)

### 2.2 Mutant and complementation vector construction

Insertion sites for the *clcA* gene (1419 bp) were predicted using the TargeTron™ algorithm (Sigma‐Aldrich) as described in Rodriguez et al. (2009). The 534|535 sense insertion location was chosen (Score: 8.69, *E*‐value: 0.065) and IBS, EBS1d, and EBS2 primers containing complementary sequences to *clcA* were used to retarget the parent TargeTron™ plasmid pKEK1140, generating pJFP1004 (Table 1). For electroporation of *F. tularensis*, electro‐competent cells were prepared as described in Rodriguez et al. (2009). Prepared cells were transformed with 0.5–1.0 μg TargeTron™ plasmid DNA at 2.5 kV, 600 Ω and 25 μF (Millipore Sigma). Transformed cells were immediately resuspended in 1 ml pre‐warmed MHII broth and recovered for 4 h at 30°C with shaking. Cells were plated on MHII agar +5% (v/v) FBS containing 10–50 μg/ml kanamycin (Calbiochem) and grown at 30°C for 4–5 days. Single colonies were streaked onto fresh plates and the presence of the intron was PCR‐verified with gene‐ and intron‐specific primers (Table A1). Confirmed insertional mutants were restreaked on MHII agar +5% (v/v) FBS without antibiotic and grown at 37°C for 2 days to cure the plasmid. Plasmid curing was confirmed via PCR (Table A1). To generate the ClcA *E. coli* complementation plasmid used in acid challenge assays, pFTClcA, WT type B *clcA* was synthesized into pEZ (Epoch Life Sciences) and inserted into the low‐copy plasmid pWSK29 (Wang & Kushner, 1991) at BamHI and XhoI sites to form the pFTClcA complementation plasmid. Expression was confirmed using western blot.

### 2.3 Cell culture and infection assay

J774A.1 murine macrophage (ATCC^®^, TIB‐67™) or THP‐1 human monocyte (ATCC^®^, TIB‐202™) cell lines were grown in DMEM or RPMI (Corning), respectively, supplemented with L‐glutamine, HEPES, 2‐Mercaptoethanol (Sigma), and 10% heat‐inactivated FBS (Atlanta Biologicals). For macrophage infections, treated 24‐well plates (Corning) were seeded with 2 × 10^5^ macrophages per well and grown to confluence. THP‐1 monocytes were differentiated in 200 nM phorbol 12‐myristate 13‐acetate (PMA, Sigma‐Aldrich) for 3 days followed by 3–5 days in fresh RPMI as described previously (Chanput et al., 2014; Daigneault et al., 2010). Infection assays were adapted from Elkins et al. (2011). Briefly, macrophages were primed with 50 ng/ml LPS‐EB (InvivoGen) for 6 h, washed with HBSS (Invitrogen), and infected with WT (OR96‐0246), *clcA* mutant, or attenuated (LVS) type B strains at a multiplicity of infection (MOI) of 50 (confirmed by plating). Plates were spun for 5 min at 350 *g* to synchronize the infection and incubated at 37°C for 1.5–2 h to allow phagocytosis to occur. Cells were washed with HBSS and left in 1 ml of tissue complete media containing 100 μg/ml gentamicin sulfate (Corning) for no longer than 45–60 min. Cells were washed again as above and replenished with 0.5 ml media containing 10 μg/ml gentamicin sulfate for the remainder of the experiment. LPS‐primed uninfected macrophages serve as negative controls. Positive controls were stimulated with TLR‐2 agonist Pam_3_CSK_4_ (300 ng/ml, InvivoGen).

### 2.4 Macrophage harvest and determination of intracellular replication

At 4‐, 18‐, and 36‐h post‐infection, 400 μl supernatant was removed from each well and stored at −80°C. 10 μl of 10× proteinase inhibitor cocktail (Roche) and 100 μl of NP‐40 lysis buffer (Alfa Aesar) were added to the remaining 100 μl/well. Cells were mechanically lysed using a 1 ml needleless syringe stopper (BD Difco) and collected in safe‐lock 2 ml microcentrifuge tubes (Eppendorf). Serial dilutions were prepared from lysates in PBS and spot plated in triplicate on MHII agar and grown at 37°C + 5% CO_2_ for 48 h. Each spot was counted; the limit of detection (LOD) = 1 colony‐forming unit (CFU). Values were back‐calculated to get to the original dilution. Dilutions with only one of the triplicate spots yielding CFU were reported as 0. Values were transformed +1 to bring values of 0 to 1 and were then log‐transformed. Log‐transformed values were averaged across experiments. Uninfected control wells were monitored for cross‐contamination.

### 2.5 RNA extraction and real‐time quantitative reverse transcription PCR (qRT–PCR)

Supernatants were removed from RNA‐dedicated wells and plates were flash‐frozen at −80°C or stored in RNA*Later™* (Invitrogen Ambion) at 4°C. Following the addition of 250 μl Isol‐RNA (5 Prime), cells were harvested by scraping as above and immediately processed, or stored at –80°C. To extract RNA, 50 μl of chloroform per 250 μl of Isol‐RNA was added, and tubes were spun at 12,000 *g* for 15 min. The top aqueous layer was removed into a new tube containing 400 μl isopropanol and stored at −20°C for at least 1 h. Tubes were spun as above and 500 μl of 75% ethanol was added to the pellet. After a final spin, the supernatant was removed, and pellets were dried before resuspending in HyClone Molecular Biology Grade Water (GE). RNA quality and quantity were analyzed using NanoDrop™ and Qubit™ (ThermoFisher), respectively. RNA was run on 1% agarose to confirm quality. RNA was reverse transcribed using the Thermo Maxima H Minus cDNA Kit with DNase and stored at −80°C. cDNA was analyzed using SYBR green (perfeCTa^®^ SYBR^®^ Green FastMix^®^ Low ROX, Quanta Biosciences) on the 7500 Fast System (Applied Biosystems). Primers were designed to yield amplicons between 50 and 150 bp (Invitrogen or IDT), and correct amplicon‐melting temperatures (*T*_m_) were verified for each primer/cDNA mix and after each run (Table A1). Primer efficiencies were determined by plotting triplicate Ct values versus log (ng cDNA template) across a 6‐series dilution (0.02–50 ng) and calculating the slope and R coefficient for each primer set, where the R coefficient corresponds to the primer efficiency (Bookout et al., 2005). Efficiencies are as follows: Human: β‐actin, 99%, Interleukin (IL)‐18, 98%, TNF‐α, 96%, IL‐1β, 99%; Murine: β‐actin, 99%, IL‐18, 99%, IL‐12, 98%, TNF‐α, 96%, IL‐1β, 99%, IL‐6, 99%. RT‐minus‐negative controls ruled out contaminating genomic DNA. No‐template controls were included in each run and did not generate primer‐specific melt curves.

### 2.6 Immunoassays

Inactivated supernatants from human and murine macrophage assays were analyzed via platinum or instant ELISA kits (eBioscience) with duplicate wells for each sample. Absorbance values (490 nm) from experimental wells were converted to concentrations using known standards and reported as averages. Absorbance values that gave negative concentrations (equivalent to blank wells) are reported as zero.

### 2.7 Macrophage cell death

96‐well plates (Corning) were seeded with a previously determined optimal concentration of 5 × 10^4^ human or murine macrophages per well and grown overnight. Cells were infected with either WT, LVS, or *clcA* mutant, and time course assays were performed as described above. At 4‐, 18‐, and 36‐h post‐infection, 50 μl of supernatant was transferred to a clean 96‐well plate. Cell death was determined using the CytoTox 96^®^ Non‐Radioactive Cytotoxicity Assay kit (Promega; Promega) and absorbance values were converted into percentages using the formula [(Experimental − Effector Spontaneous − Target spontaneous)/(Target maximum − Target spontaneous)] × 100.

### 2.8 Acid Challenge

The acid challenge was performed as described previously (Castanie‐Cornet et al., 1999; Iyer et al., 2002). Wild‐type *E. coli* MG1655, double knockout MG1655Δ*clcA*Δ*clcB* (generously gifted from Chris Miller), and MG1655Δ*clcA*Δ*clcB* complemented with pFTClcA or pWSK29 empty vector control were grown overnight in LB supplemented with 20 mM glucose, and 100 μg/ml ampicillin where appropriate. Overnight cultures (17–20 h) were OD‐normalized, and 10 μl were incubated in the acid challenge buffer (40 mM KCl, 80 mM KH_2_PO_4_, 33 mM H_3_PO_4_, 1.7 mM sodium citrate, 20 mM glucose, pH 2.5) alone and with 1.5 mM glutamate (Spectrum^®^) for 2 h at 37°C without shaking. After 2 h, survival was determined by plating, compared to initial baseline counts, and graphed as percent survival. pFTClcA and pWSK29 empty vector were induced 2 h before the acid challenge with 2.5 mM IPTG.

### 2.9 Statistics

Data were imported into GraphPad Prism version 6.0+ for Mac OS X, GraphPad Software, La Jolla California USA, www.graphpad.com. Fixed two‐way ANOVA with Tukey's post‐test was used to compare the intracellular growth of *Francisella* strains since it was determined that no significant variation exists across experiments. Colony‐forming units (CFU) were log‐transformed and averaged for each dilution series. Gene expression data were analyzed by a mixed‐effects model with the Tukey test to correct for multiple comparisons. Acid challenge experiments were analyzed using the Kruskal–Wallis non‐parametric test and corrected for multiple comparisons using Dunn's test.

## 3 RESULTS

### 3.1 Human and murine macrophage cell lines are permissive for the growth of WT type B

**FIGURE 1:**
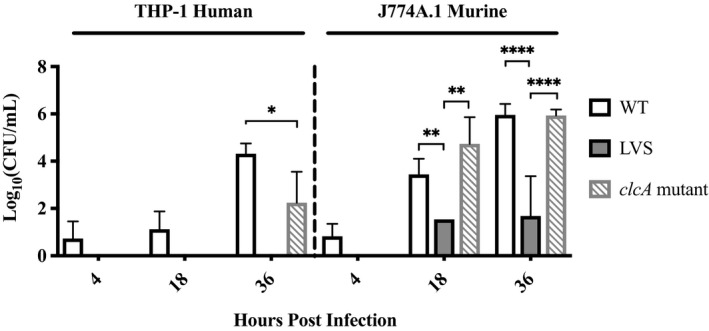
Primed human and murine macrophages are permissive for replication of WT type B, while *clcA* mutant shows delayed replication. Primed human THP‐1 (left) or primed murine J774A.1 (right) macrophages were grown to confluence in 24‐well plates and infected with virulent type B strain OR96‐0246 (WT), *clcA* mutant, or LVS as described in Materials and Methods. After phagocytosis, cells were washed and treated with gentamicin to remove extracellular bacteria. At 4‐, 18‐, and 36‐h post‐infection, macrophages were lysed to release intracellular bacteria. Bacteria were plated on MHII agar and incubated for 48 h at 37°C with 5% CO_2_ before enumeration. Growth is reported as log_10_ (CFU/ml) over time. Data represent four independent experiments for WT and two independent experiments for LVS and *clcA* mutant. Treatments were performed in triplicate or quadruplicate, with triplicate spot plating. Counts were averaged for each dilution. Bars represent standard error of the mean (SEM). *p < 0.05; **p < 0.01; ****p < 0.0001 as determined by two‐way ANOVA (Table 2). Comparisons to LVS in human macrophages were not performed since no CFU were recovered

**FIGURE A2:**
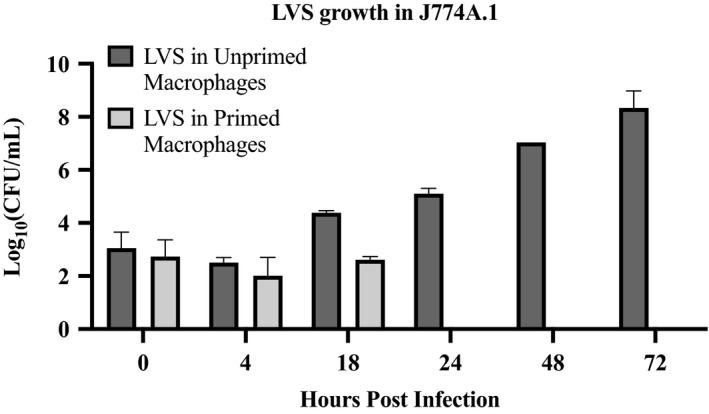
LVS growth is determined by the activation state of murine macrophages. J774A.1 murine macrophages were primed with LPS‐EB or left untreated and infected with LVS as described in Materials and Methods. An MOI of 1:150 CFU:macrophage was used. After phagocytosis, cells were washed and treated with gentamicin to remove extracellular bacteria. At 4, 18, and 36 hours post‐infection (HPI) macrophages were lysed to release intracellular bacteria. Bacteria were plated on MHII agar and incubated for 48 h at 37°C with 5% CO_2_ before enumeration. Growth is reported as Log_10_(CFU/ml) over time. Representative data are shown. Treatments were performed in triplicate, with triplicate spot plating. Counts were averaged for each dilution. Bars represent standard error of the mean (SEM)

A better understanding of how virulent type B strains behave in macrophages is required to further explore the attenuating mechanisms of LVS. The tularemia mouse model has its limitations for predicting effects in humans, as mice are overly susceptible to *F. tularensis* infection (Ray et al., 2010). This is demonstrated by the fact that at some doses LVS retains virulence in mice. However, successful *Francisella* infection generally correlates with its ability to replicate within macrophages, its primary reservoir (Bhatnagar et al., 1994). Relative to type A strains, few studies have investigated the intracellular infection kinetics of virulent type B strains. To compare the in vitro growth of WT type B to that of its attenuated counterpart, primed murine and human macrophage cell lines were infected with OR96‐0246 or LVS at an MOI of 50. Primed macrophages were used due to previous optimization studies examining LVS replication in primed versus unprimed macrophages (Figure A2), and due to the observation by Netea et al. (2009) that, in contrast to monocytes, macrophages (including THP‐1 cells) require two stimuli for IL‐1β release. Similar to what others have shown for primed macrophages, human and murine macrophage cell lines rapidly controlled intracellular LVS replication (Figure 1; Bosio & Elkins, 2001). In human macrophages, recovered LVS CFU were below the LOD at 4‐h post‐infection (HPI), with no CFU recovered after that. Initially, no LVS CFU were recovered from murine macrophages, with average counts increasing to approximately 1 log CFU/ml by 18 HPI, which remained steady at 36 HPI. In contrast, average WT type B counts by 36 HPI reached 4.3 and 6.0 log CFU/ml in human and murine macrophages, respectively, consistent with increased susceptibility of mice compared to humans. Recovered WT type B CFU were statistically different from LVS CFU at multiple time points (Table 2). From these data, we conclude that OR96‐0246 is capable of robust replication inside macrophages, consistent with what has been observed for other WT type B strains. Thus, intracellular growth is a distinguishing factor between WT and attenuated type B strains.

**TABLE 2:** *p*‐values for significant differences in replication between WT, LVS, & *clcA* mutant

Treatment compared	Host cell type	Hours post‐infection	*p*‐value
WT versus *clcA* mutant	Human	36	<0.05
WT versus LVS	Murine	18	<0.01
LVS versus *clcA* mutant	Murine	18	<0.005
WT versus LVS	Murine	36	<0.0001
LVS versus *clcA* mutant	Murine	36	<0.0001

^a^ Comparisons to LVS in human macrophages were not performed since no CFU were recovered.

### 3.2 A proton:chloride antiporter mutant shows delayed proliferation inside macrophages

**FIGURE A3:**
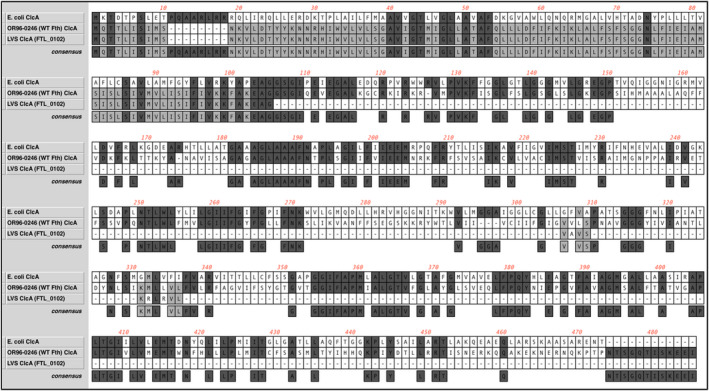
MacVector protein alignments of *Francisella* (WT and LVS) and *Escherichia coli* and chloride transporter proteins. The consensus is shown in gray. Important conserved residues are serine (107), glutamic acid (148, 203), and tyrosine (445). LVS truncation occurs at position 106 resulting in an early stop codon, producing only 20% of the full‐length protein

**FIGURE A4:**
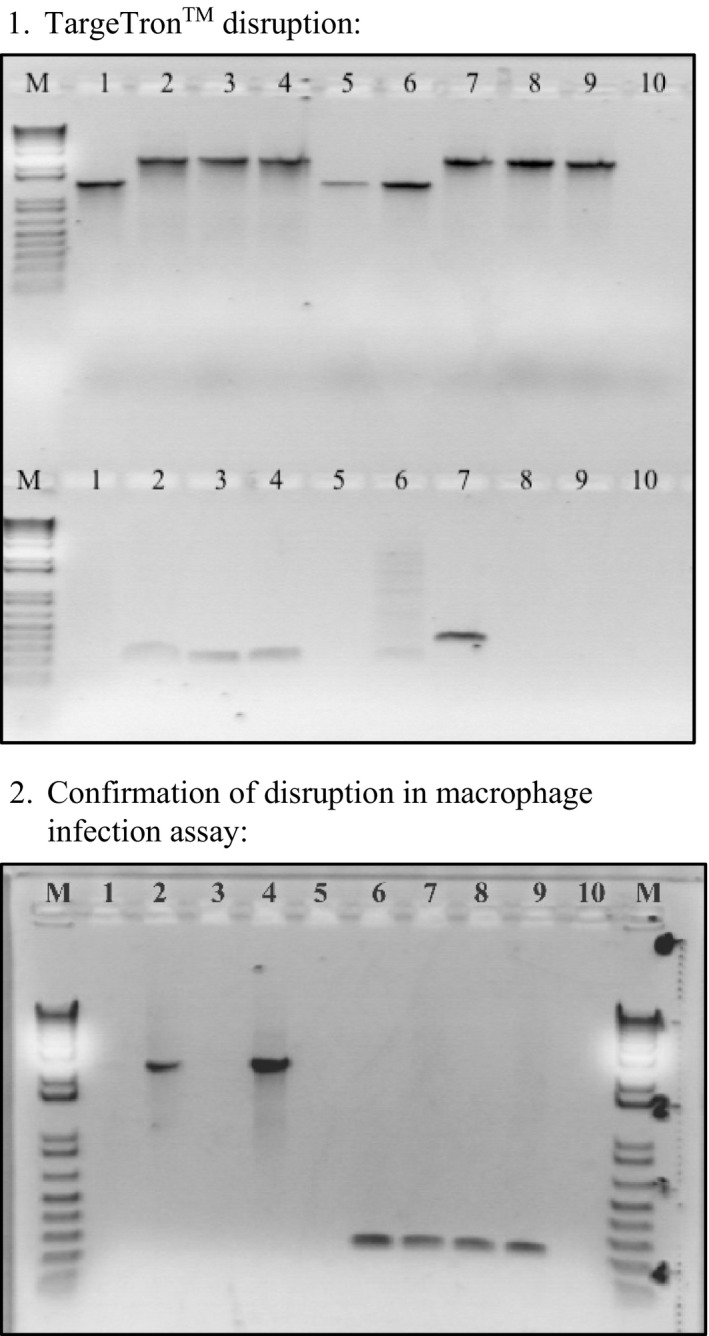
Confirmation of *clcA* disruption. Gel 1, Top: TargeTron™ colony PCR products from electroporation of OR96‐0246 with pJFP1004 to generate *clcA* mutant. M, marker. Gene‐specific 0095 F/R primers: Lane 1, WT control; Lanes 2–4, transformants; Lane 5, NTC. Duplicate PCR reaction run on Lanes 6–10 to verify results. Band in Lane 5 (NTC) is likely an artifact from colony PCR/gel loading and is confirmed negative in Lane 10 (NTC). Gel 1, Bottom: Lanes 1–5, same as Lanes 1–5 above using intron‐specific F/R primers. Lanes 7–10 are products from Kan probe F/R primers to confirm plasmid curing: Lane 7, Kan^R^ positive control; Lanes 8–10, transformants restreaked on Kan^‐^ MHII plates; Lane 11, NTC (partially cropped). Gel 2: Colony PCR of recovered CFU from *clcA* mutant‐infected wells at 36 HPI were run on 1% agarose confirming the presence of disruption. Lanes 1 and 3, supernatants from J774A.1 and THP‐1 wells, respectively; Lanes 2 and 4, colonies from plated J774A.1 and THP‐1 lysates; Lane 5, NTC (all with gene‐specific 0095 F/R primers). Lanes 6–9, same as Lanes 1–4 but with intron‐specific F/R primers; Lane 10, NTC

**FIGURE A5:**
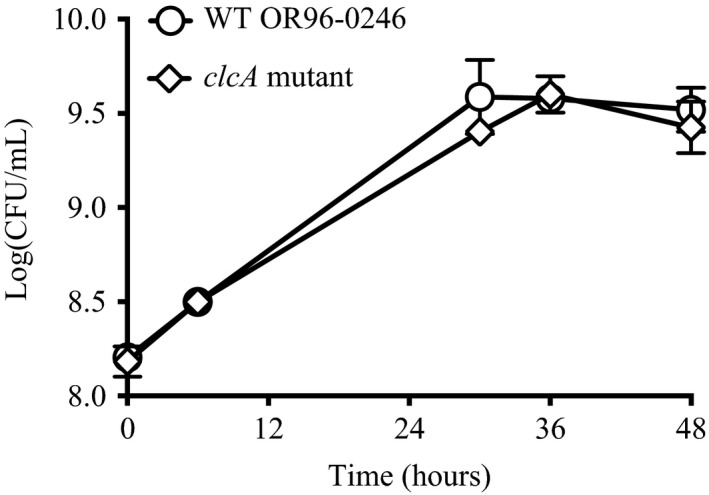
Growth of *clcA* mutant matches that of WT OR96‐0246 in broth culture. WT and *clcA* mutant strains were grown in modified Mueller‐Hinton broth as described in Materials and Methods. Data represent two independent experiments with standard deviation shown

We next sought to determine the individual roles of candidate genes in LVS attenuation identified by our lab and others through comparative genomics (Petrosino et al., 2006; Rohmer et al., 2006). Upon the prioritization of candidate genes, we focused our efforts on the proton:chloride antiporter encoded by *clcA*. ClcA is part of the conserved CLC family of chloride channels and transporters with isoforms spanning prokaryotes and eukaryotes (Miller, 2006). In LVS, a single base‐pair deletion in *clcA* caused a frameshift mutation resulting in an early stop codon in the gene. The outcome was a marked truncation of ClcA (loss of 80% of the full‐length protein), which is predicted to have a deleterious impact on protein function (Rohmer et al., 2006). Notably, conserved residues important for protein's function in *E. coli* occur after truncation, further supporting ClcA is non‐functional in LVS (Figure A3). To study the role of ClcA in *Francisella* independently of LVS, we used the TargeTron™ mutagenesis system to create a chromosomal disruption in OR96‐0246 *clcA* (*clcA*::*ltrB*_Ll_; hereafter *clcA* mutant) (Refer to Table 1, Materials and Methods, and Figure A4). Previously adapted for use in *Francisella*, this system uses a *Lactococcus lactis* group II intron with an associated ribonucleoprotein complex (RNP) and a native *Francisella* promoter (Rodriguez et al., 2008, 2009). *ClcA* is not predicted to be part of an operon, assuaging concerns of polar effects. To determine whether this disruption is partly responsible for LVS attenuation, we repeated the above macrophage infection assay with the *clcA* mutant and observed a delay in replication (Figure 1). In infected human macrophages, no *clcA* mutant CFU were recovered (or were below the LOD) until 36 HPI, and bacterial counts were significantly lower than those of WT by an average of 2 log CFU/ml. A shorter replication delay was observed in murine macrophages, with 4.7 log CFU/ml recovered at 18 HPI, and 5.9 log CFU/ml by 36 HPI (Figure 1). An intermediate phenotype was expected for *clcA* mutant since it is the result of a single gene disruption and not dozens in the case of LVS. Indeed, recovered *clcA* mutant CFU were statistically different compared to LVS CFU at multiple time points, but not WT (Table 2). In summary, *clcA* mutant reached WT levels by 36 HPI in murine macrophages but remained impaired in human macrophages (Table 2). While the TargeTron™ gene knockout system is specific and permanent, *clcA* mutant bacteria were collected from macrophages 36 HPI to confirm the continued presence of the gene disruption and that replication was not due to escape mutants (Figure A4). Meanwhile, no differences were observed between WT and *clcA* mutant when grown in broth culture (Figure A5). Thus, a reproducible trend emerged that *clcA* mutant displays a lag in intracellular growth that is not observed in broth culture, which may have significant consequences in the context of innate immune detection and clearance by macrophages.

### 3.3 Immune activation profiling highlights differences in WT‐infected macrophages compared to attenuated strains

**FIGURE 2:**
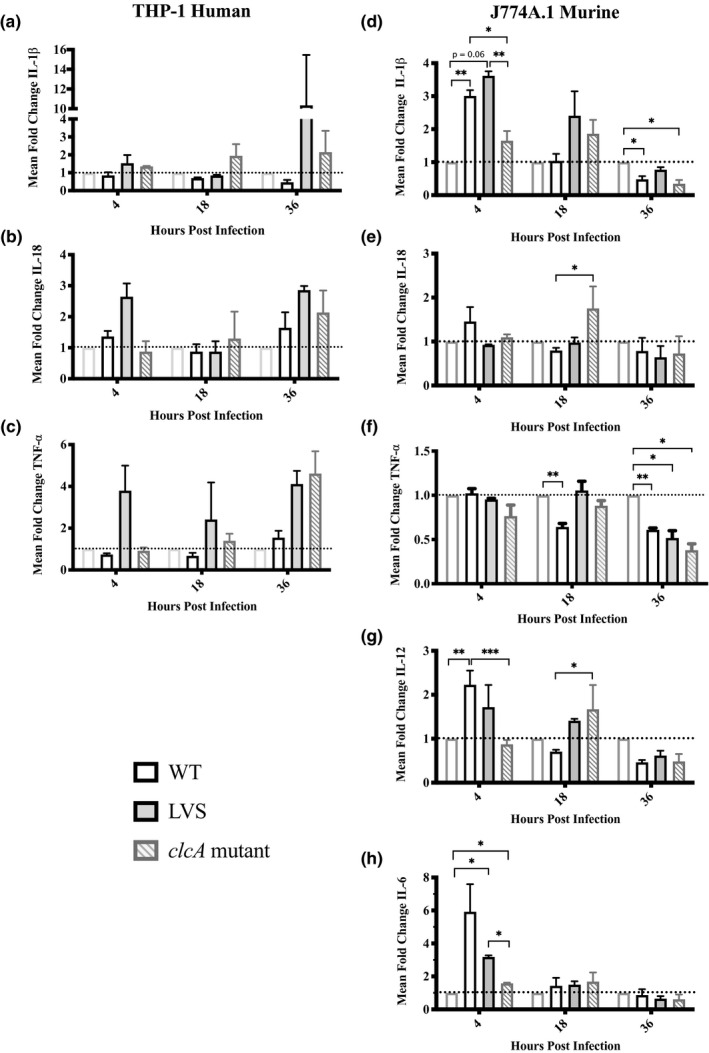
Pro‐inflammatory cytokine gene expression profiles in macrophages reflect the virulence status of strains. Total RNA from infected macrophages was collected, reverse‐ transcribed, and analyzed via qRT‐PCR for human (a–c) and murine (d–h) macrophages as described in Materials and Methods. Fold change is normalized to β‐actin. Samples are calibrated to transcript levels from uninfected macrophages, represented by the dashed line at 1. Means and SEM are shown. Samples were tested in duplicate for each gene with technical triplicates. Human data represents one independent experiment, and no significant differences are reported. Murine data represents two independent experiments each with two technical replicates. *p < 0.05; **p < 0.01; ***p < 0.001; ****p < 0.0001 as determined by two‐way ANOVA

Activated macrophages secrete pro‐inflammatory cytokines in response to intracellular pathogens. IL‐1β, IL‐6, and TNF‐α are important for acute phase protein production and fever induction, while IL‐12, IL‐18, and IFN‐γ recruit neutrophils to the site of infection and elicit a T helper type 1 (Th1) adaptive immune response. Recent studies describe the ability of virulent type A strain Schu S4 or type B strain FSC200 to suppress inflammatory cytokine transcripts in human monocytes and mouse macrophages, respectively, upon coinfection with avirulent *F. novicida* (Bauler et al., 2014; Gillette et al., 2014). To explore differences in the ability of WT type B, LVS, and *clcA* mutant to modulate cytokine gene expression, we employed real‐time quantitative reverse transcription PCR (qRT–PCR). Using the comparative Ct method, we compared transcript levels of infected macrophages to those of uninfected macrophages, normalized to the β‐actin gene (Figure 2). In human macrophages, gene expression levels for IL‐1β, IL‐18, and TNF‐α from WT infections remained low and were never two‐fold higher than uninfected macrophages (Figure 2A–C). In contrast, LVS‐infected macrophages showed a three‐ to four‐fold increase in TNF‐a and IL‐18 expression levels compared to uninfected controls at almost every time point (except 18 HPI for IL‐18), suggesting that macrophages are in a state of immune activation even after replication has been controlled, or that LVS infection may activate macrophages earlier than less attenuated strains. The greatest change in gene expression of LVS‐infected macrophages was a ten‐fold increase at 36 HPI for IL‐1β. For macrophages infected with *clcA* mutant, modest increases ≤ two‐fold were observed at 18 HPI for IL‐1β, IL‐18, and TNF‐a, but rose to two to five times greater than uninfected controls at 36 HPI, coinciding with recoverable CFU. Due to low primer efficiencies, IL‐6 and IL‐12 were not examined for human macrophages.

A different trend emerged in infected murine macrophages when we compared cytokine expression levels (Figure 2D–H). Both WT‐ and LVS‐infected macrophages initially showed increased levels of IL‐1β, IL‐12, and IL‐6 compared to uninfected controls at 4 HPI. However, the expression of these cytokines in WT‐infected macrophages subsequently declined to at or below those of uninfected controls at all later time points tested, suggesting that WT type B may actively suppress cytokine‐related responses. These results are in agreement with observations made by Bauler et al., who showed that IL‐1β, IL‐6, and CXCL1 mouse macrophage transcript levels were reduced upon infection with virulent type B strain FSC200 (Bauler et al., 2014). Conversely, IL‐1β and IL‐12 expression levels in LVS‐infected macrophages were higher than uninfected controls at 4 and 18 HPI. Cytokine expression levels for IL‐1β, IL‐18, and IL‐12 in murine macrophages infected with *clcA* mutant were highest at 18 HPI, with an almost two‐fold increase compared to uninfected controls. IL‐6 was only slightly elevated in *clcA* mutant compared to uninfected controls. TNF‐a expression levels did not vary greatly among WT, LVS, or *clcA* mutant, but were reduced by half compared to uninfected controls at 36 HPI. Intriguingly, expression levels for all cytokines examined were decreased for all treatments at 36 HPI.

**FIGURE A6:**
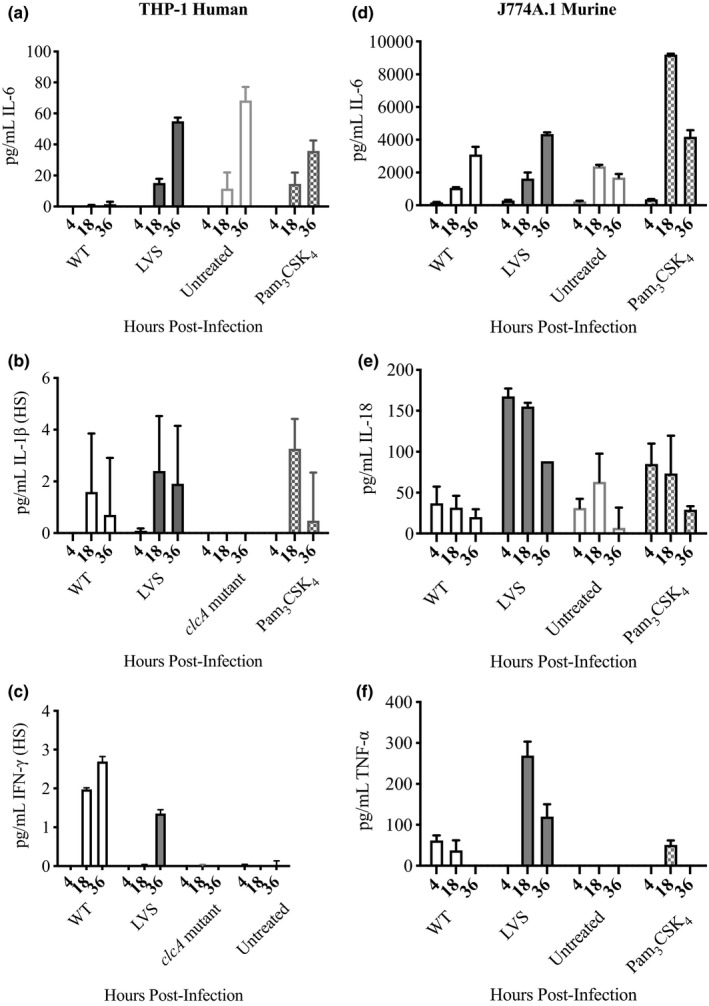
Cytokine profiling shows diminished secretion of pro‐inflammatory cytokines by primed murine macrophages when infected with WT compared to LVS, and for IL‐6 in human macrophages. Primed human (A‐C) or J774A.1 murine (D‐F) macrophages were infected with WT, LVS, and *clcA* mutant (B and C only) as described in Materials and Methods. Supernatants were inactivated and analyzed by ELISA for the indicated inflammatory cytokines. Murine IL‐6 values in panel D are as follows: WT means at 4, 18, and 36 HPI are 179, 1064, and 3107 pg/ml, respectively; LVS means at 4, 18, and 36 HPI are 291, 1632, and 4351 pg/ml, respectively; Untreated means at 4, 18, and 36 HPI are 239, 2381, and 1688 pg/ml, respectively; Pam_3_CSK_4_ means at 4, 18, and 36 HPI are 360, 9206, and 4187 pg/ml, respectively. HS—high sensitivity. Values below the limit of detection are reported as zero. Mean with SEM shown for two independent experiments. Pam_3_CSK_4_ is a TLR2 agonist and serves as a positive control

Initial experiments to compare murine cytokine secretion profiles among strains by ELISA revealed lower levels of the pro‐inflammatory cytokines IL‐6, IL‐18, and TNF‐α for WT‐infected macrophages compared to LVS, suggesting that the innate immune response helps stratify virulent and attenuated strains in vitro (Figure A6A‐C). In human macrophages, IL‐6 was not detected in WT infection supernatants (Figure A6D). Low levels of human IL‐1β and TNF‐α were recovered from WT, LVS, and *clcA* mutant infection supernatants, but were not considered physiologically relevant (<10 pg/ml for all strains, Figure A6E‐F). Due to the lack of reproducibility acquiring positive signals from subsequent supernatants, and the observation that cytokine gene expression was decreased in all murine infection treatments at 36 HPI, we instead chose to study intracellular infection kinetics and immune suppression by examining host cell death.

**FIGURE 3:**
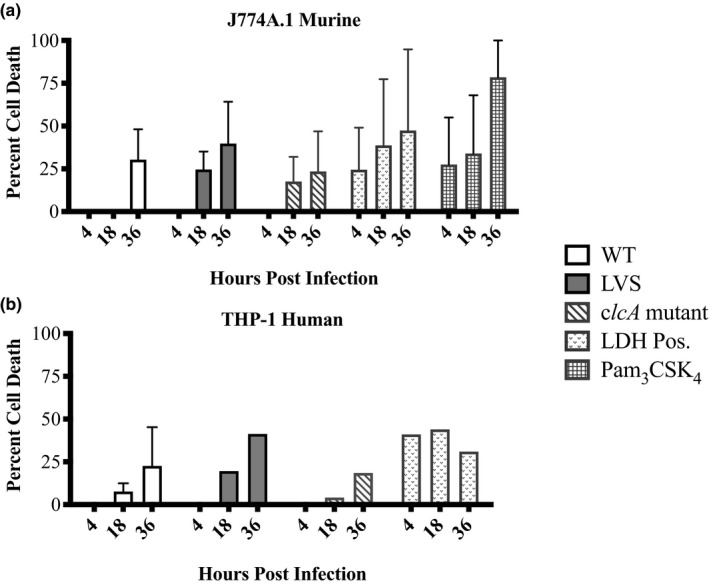
Cell death of infected macrophages. 96‐well plates were seeded with 5 × 10^4^ J774A.1 murine (a) or THP‐1 human (b) macrophages and infected with either WT type B, attenuated type B strain LVS, or *clcA* mutant in quadruplicate. At the indicated time points supernatants were transferred to a new plate and cell death was determined by the lactate dehydrogenase assay as described in Materials and Methods. Absorbance values were averaged and converted into percentages. A representative experiment is shown for *clcA* mutant and human macrophage cell death. Two independent experiments are shown for WT‐ and LVS‐infected murine macrophages. Mean and SEM shown. No significant differences were detected

**FIGURE A7:**
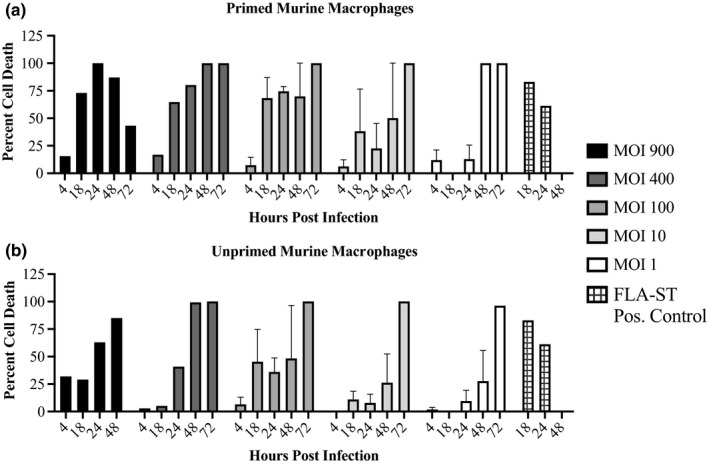
LVS elicits cell death in a dose‐dependent manner in murine macrophages. Primed (a) or unprimed (b) murine macrophages were infected with LVS at five different MOIs. At the indicated time points, supernatants were transferred to a new plate and cell death was determined by the lactate dehydrogenase assay as described in Materials and Methods. Absorbance values were averaged and converted into percentages. Mean and SEM shown for two independent experiments

We hypothesized that the fate of the macrophage depends on the strain used to infect the cell. Previous cell‐death assay optimization studies showed a dose‐dependent response to LVS infection, with cell death consistently seen by 18 HPI in primed macrophages (40%–70%, Figure A7). Using the lactate dehydrogenase (LDH) assay, we measured cell death upon infection with WT, LVS, or *clcA* mutant ( Promega, 2009 ) (Figure 3). While not statistically significant, we observed that murine macrophages infected with attenuated strains LVS or *clcA* mutant underwent cell death at least 18‐h earlier than those infected with WT type B. Cell death from human macrophages was observed for all strains at 18 HPI: LDH levels were 12% and 18% higher in LVS than in WT infection supernatants at 18 and 36 HPI, respectively, while similar LDH levels were observed between WT and *clcA* mutant infection supernatants. These data, combined with cytokine gene expression and preliminary secretion data, support the hypothesis that LVS, and to a lesser extent *clcA* mutant, is detected by the host and triggers an inflammatory response, resulting in cell death. In contrast, WT type B continues to replicate inside macrophages, suggesting that WT type B may actively suppress the macrophage innate immune response. In support of these findings, lack of murine macrophage cell death 12 HPI with virulent type A strain Schu S4 was previously reported (Bauler et al., 2014). Finally, the decrease in gene expression levels at 36 HPI for all strains may be a result of increasing macrophage cell death at 36 HPI.

### 3.4 *Francisella* ClcA functionally complements acid resistance in a *clcA*‐deficient *E. coli* strain

From the observed delay in replication of *clcA* mutant in macrophages, we postulated that the primary activity of ClcA occurs early on during the intracellular lifecycle of *Francisella*. Following phagocytosis, virulent *F. tularensis* strains readily escape the phagosome and replicate in the host cytosol. A prolonged but similar level of escape is observed between LVS and *F. novicida* strain U112, which does not cause disease in immunocompetent humans (Chong et al., 2008; Golovliov et al., 2003). However, the environment of the *Francisella*‐containing phagosome (FCP) before phagosomal escape is subjected to controversy. Clemens et al. report that many phagosomes containing live *F. tularensis* do not stain for the host vacuolar ATPase within the first 3 h of infection, and treatment with bafilomycin A (BFA) does not affect the phagosomal membrane disruption (Clemens et al., 2004, 2009). Conversely, Santic et al. have evidence to support the acidification of FCPs within 15‐min post‐infection and show that BFA treatment rapidly blocks phagosomal escape by *Francisella* (Santic et al., 2008). Others still have shown a mere delay in phagosomal escape following treatment with BFA or concanamycin A (Chong et al., 2008). These studies used different strains, cell types, modes of phagosomal uptake, and stringencies for defining phagosomal disruption, all of which could be contributing factors to these contradictory results. We therefore chose to study ClcA in the context of the extensively studied model system of *E. coli*.

In *E. coli*, the H^+^/Cl^−^ antiporter ClcA (previously EriC) functions in the context of extreme acid resistance (XAR). Bacteria counteract low extracellular pH through decarboxylation‐linked proton utilization of imported charged amino acids (glutamate and arginine at pH 2.5, and lysine and ornithine at higher pH) (Foster, 2004; Iyer et al., 2003). In *E. coli*, ClcA acts as an electrical shunt to reverse hyper‐polarization of the bacterial membrane that occurs through the proton‐consuming decarboxylation process (Foster, 2004). *E. coli* possesses a second redundant homolog known as *clcB* (previously *mriT*), and only when both genes are deleted is a phenotype observed (Iyer et al., 2002). While ClcA and ClcB in *E. coli* are believed to have different pH optima (Chris Miller, personal correspondence), there is no redundant gene in *Francisella*. Several *F. tularensis* type B strains, including OR96‐0246, were previously examined for gastric acid resistance, but may not appropriately reflect conditions during a macrophage infection (Adcock et al., 2014). Furthermore, while reductions in viability were observed for cultures exposed to SGF (synthetic gastric fluid, pH 2.5 and 4.0) compared to acidic PBS (pH 2.5), survival was nonetheless higher for WT strains than for attenuated strains such as LVS. Initial experiments to determine acid sensitivity in WT, LVS, and *clcA* mutant revealed that LVS was indeed sensitive to acid challenge; however, no differences were observed between WT and *clcA* mutant. Our best explanation is that additional protein(s) is (are) disrupted in LVS compared to the *clcA* mutant that causes LVS to be more sensitive to acid in vitro, but as stated above, these in vitro experiments may not mirror conditions inside macrophages.

**FIGURE 4:**
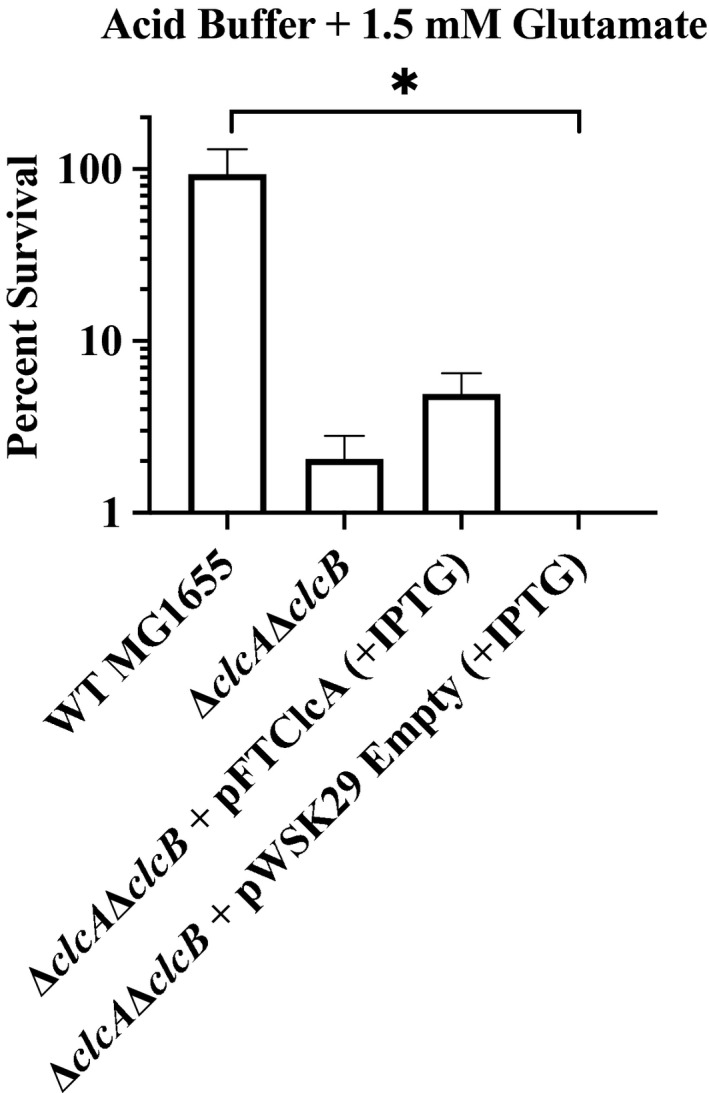
WT *Francisella* type B ClcA can partially restore acid tolerance in an acid‐sensitive *E. coli* mutant. WT *E. coli* strain MG1655, double mutant Δ*clcA*Δ*clcB*, or double mutant complemented with pFTClcA or pWSK29 empty vector control (both induced with 2.5 mM IPTG) were plated for survival after incubation for 2 h in acid buffer with 1.5 mM glutamate at pH 2.5. Counts were normalized to PBS input controls, and survival ratios were converted into percentages. For all strains, survival in the absence of glutamate was less than 0.05%. Data represents 4 to 6 independent experiments, with averaged triplicate CFU counts. Mean with SEM shown. *p < 0.05 as determined by the Kruskal–Wallis test with Dunn's post‐test to adjust for multiple comparisons

We therefore sought to determine whether *Francisella* ClcA could functionally complement the *E. coli* MG1655Δ*clcA*Δ*clcB* double mutant strain under acidic conditions. Overnight cultures of WT MG1655, MG1655Δ*clcA*Δ*clcB*, and MG1655Δ*clcA*Δ*clcB* complemented with either inducible plasmid pFTClcA or empty vector pWSK29 were added to acid challenge buffer (ACB, pH 2.5) alone or supplemented with 1.5 mM glutamate, and survival was measured after 2 h. WT *E. coli* displays only modest survival in ACB alone, but when grown in the presence of glutamate exhibits increased survival (Figure 4). In contrast, MG1655Δ*clcA*Δ*clcB* does not recover even with amino acid supplementation, presumably due to a buildup of charge across the membrane that eventually halts the coupled amino acid exchange system (Garcia‐Celma et al., 2013). However, upon the complementation of Δ*clcA*Δ*clcB* with pFTClcA, acid challenge survival was restored by a modest 5%–10%. These levels are similar to those previously shown (10%–30%) for complementation in *E. coli* (Iyer et al., 2002 ), but may be lower due to less efficient protein localization. In contrast, the Δ*clcA*Δ*clcB* strain complemented with empty vector control did not recover. While further studies are required to determine if ClcA plays a role specifically within *Francisella*‐containing phagosomes, our results raise the possibility that ClcA contributes to acid survival in *Francisella* and warrants further investigation.

## 4 DISCUSSION

To date, LVS remains the current gold standard for protection against *F. tularensis* in humans (El Sahly et al., 2009; Hornick & Eigelsbach, 1966; McCrumb, 1961). Myriad new experimental killed, subunit, and live attenuated vaccine candidates have emerged within the past decade. However, variability in animal models, complex vaccination routes and regimens, and increased safety risk due to possible reversion are all significant challenges to overcome (Conlan, 2011; Marohn & Barry, 2013; Shen et al., 2004). Until recently, WT type B strains were overlooked for live attenuated vaccine development. Attempts to find the attenuation “sweet spot” in type A strains, which must balance attenuation and viability, remain unsuccessful. Such attempts either result in over‐attenuation, and therefore loss of protection, or pose a safety risk (reviewed in Jia & Horwitz, 2018). Exemplifying this point is the progression of one candidate mutant, *clpB*, which was initially engineered in virulent type A strain Schu S4 and was shown to surpass LVS efficacy against intranasal challenge with more than 50 CFU Schu S4 in mice (providing 80% or greater protection) (Conlan et al., 2010; Rockx‐Brouwer et al., 2012; Ryden et al., 2013; Shen et al., 2010; Twine et al., 2012; Wehrly et al., 2009). A second locus was deleted due to reversion concerns, abolishing all protection. The single *clpB* gene deletion was subsequently moved into type B strain FSC200, which also out‐performed LVS, albeit not to the extent of Schu S4Δ*clpB* (Golovliov et al.,). Nonetheless, FSC200Δ*clpB* serves as proof of concept that mutants of type B backgrounds can meet both efficacy and safety standards. Notably, *clcA* is located just four genes upstream of *clpB*.

In this study, we used an in vitro model to investigate the innate immune signals that precede a Th1 response in the host (Cole et al., 2006; Cowley & Elkins, 2011). Located at primary infection sites, macrophages act as important first responders to initiate an inflammatory response (Gavrilin et al., 2006; Rivera et al., 2016). Together with the TLR‐2/MyD88/NF‐κB signaling pathway, cytosolic innate immune activation potentiates cleavage of pro‐caspase‐1 and subsequent secretion of pro‐inflammatory cytokines, resulting in pyroptosis (Atianand et al., 2011; Bergsbaken et al., 2009; Cole et al., 2007; Fernandes‐Alnemri et al., 2010; Katz et al., 2006; Man et al., 2015; Meunier et al., 2015). This host cell death effectively removes the replicative niche for *F. tularensis*, mobilizes adaptive immunity, and consequently inhibits bacterial dissemination. However, virulent strains well‐adapted to the intracellular niche employ stealth strategies to avoid host detection. This is demonstrated by our observation that WT‐infected macrophages show a delay in cell death compared to LVS and *clcA* mutant in murine macrophages. Thus, immune activation profiles of macrophages reflect the virulence status of *Francisella* strains.

These findings contribute to the limited knowledge of type B pathogenesis and serve as a benchmark to compare to LVS and other attenuated type B strains for further development of vaccine candidates. Consistent with what others have found, WT type B replicates to greater numbers in primed macrophages compared to LVS in both human and murine macrophages (Ray et al., 2010). While LVS replication was lower here than what other studies have observed, likely due in part to the activated state of our macrophage cell lines (Figure A2), WT strain OR96‐0246 was nonetheless capable of robust replication. Under the same assay conditions, when the chloride transporter *clcA* was disrupted in type B, we observed a reproducible trend of delayed intracellular replication, with significantly fewer *clcA* mutant CFU recovered in human macrophages at 36 HPI. While the exact mechanism is unknown, the observation that *clcA* mutant was still capable of replication suggests that ClcA plays a role early in pathogenesis and may not influence later lifecycle stages of *Francisella*. As ClcA homologs in other Gram‐negative bacteria reside in the inner membrane, it is unlikely that the mutant is defective for steps related to macrophage entry. The J774A.1 macrophage‐like cell line is commonly used to study intracellular kinetics of *Francisella*, and LPS‐stimulated THP‐1 cells are an acceptable alternative to, and have several advantages over, PBMC macrophages (Daigneault et al., 2010; Qin, 2012). Nonetheless, this work should be further validated by studies in primary cells, as well as in rat or murine animal models, which was a limitation of our study. Lastly, one standing weakness of the current study is that we did not perform complementation studies for the *clcA* mutant in *Francisella*, but only in *E. coli*.

The importance of ion channels in *Francisella* is made clear by their identification in genetic screens, but the roles of these proteins have never been biochemically demonstrated (Meibom & Charbit, 2010). Based on studies in *E. coli*, Rohmer et al. suggested that ClcA may be important for survival at low pH (Johansson et al., 2000). As the cytosol is the site of *Francisella* replication, we hypothesized that the delay we observed for *clcA* mutant replication was a result of extended time within the *Francisella*‐containing phagosome (FCP), where chloride ions are readily available (Sonawane et al., 2002). It was previously shown that inhibition of phagosomal acidification delays *F. tularensis* Schu S4 escape into the cytosol (Chong et al., 2008). Chong *et al*. conclude that important bacterial components required for phagosomal escape may be triggered by intravacuolar pH, and the CLC family of chloride channels is activated at a low pH (Garcia‐Celma et al., 2013). Specifically, acidification directs conformational changes that activate voltage‐gated chloride transporters (Basilio et al., 2014). Our finding that *Francisella* ClcA can partially rescue an acid‐sensitive *E. coli* strain under low pH conditions offers one potential explanation for conflicting results surrounding the *Francisella*‐containing phagosome. One hypothesis is that the initial acidification upon phagocytosis is required to activate ClcA, but subsequent ClcA activity would offset acidification before phagosomal escape. In this purely speculative model, ClcA increases resistance to acidic stress or may provide an environmental cue for the expression of virulence factors required for phagosomal escape. Unfortunately, in‐depth examination of phagosomal survival and escape was outside the scope of this manuscript, as lack of funding for this project and a change in the laboratory's research focus precluded further experimentation.

The term “nutritional virulence” was recently popularized to describe the ability of a pathogen to adapt its metabolism to use nutrients available in the host (Abu, 2013; Abu Kwaik & Bumann, 2013; Santic & Abu, 2013). More specifically, the acquisition of amino acids through scavenging can be deemed a form of virulence. Ramond et al. report the existence of 11 amino transporters in *Francisella* belonging to the family of amino acid/polyamine/organocation (APC) transporters (Ramond et al., 2014). Of these, the arginine (ArgP) and glutamate (GadC) transporters were the only two repeatedly identified in four screens and recently shown to be functional amino acid transporters in *Francisella novicida* strain U112 and *F. tularensis* subsp. *holarctica* strain LVS (Kraemer et al., 2009; Maier et al., 2007; Peng & Monack, 2010; Weiss et al., 2007). In *F. novicida, gadC* was linked to oxidative stress defense and phagosomal escape (Ramond et al., 2014). The authors further demonstrated that *Francisella* GadC confers equivalent acid resistance in an *E. coli gadC* mutant (at pH 2.5, no glutamate supplementation). More recently, Ramond et al. showed that disruption of *argP* in *F. novicida* results in delayed phagosomal escape and intracellular replication (Ramond et al., 2015). The same authors also tested *argP* mutant survival under acid stress, but at pH 4. Lastly, a replication defect was also observed in LVS Δ*argP* in J774A.1 macrophages, but only after 10‐h post‐infection. Thus, these two transporters also clearly play a role during the early intracellular lifecycle of *Francisella* and may work in tandem with ClcA (Castanie‐Cornet et al., 1999). Additional biochemical evidence is needed to determine what roles, if any, these systems play in acid tolerance in the context of macrophage infections.

## 5 CONCLUSIONS

Type B infections are associated with waterborne outbreaks and are on the rise in certain parts of the world including the United States (Appelt et al., 2020; Gehringer et al., 2013; Kilic et al., 2015; Pedati et al., 2015; Tanaka et al., 2008; Yeni et al., 2020). A genetically defined, live attenuated vaccine derived from a virulent type B strain may prove effective and safe. Despite the limitations of our study, our results suggest that the disruption of *clcA* is one contributor to LVS attenuation and may be a useful candidate to target for a live attenuated vaccine against tularemia. Our laboratory has adapted the parent *Francisella* TargeTron™ plasmid for the disruption of all 17 attenuation candidate genes (Table A2), thus increasing the available tools to study the contribution of these genes to *Francisella* virulence.

## CONFLICT OF INTEREST

None declared.

## ETHICS STATEMENT

Work involving strain OR96‐0246 was performed under biosafety‐level 3 (BSL‐3) containment conditions, with all standard operating procedures approved by the Office of Environmental Safety at Baylor College of Medicine in accordance with the Centers for Disease Control and Prevention.

## AUTHOR CONTRIBUTIONS

**Lisa Matz:** Conceptualization (lead); Data curation (lead); Formal analysis (lead); Investigation (lead); Methodology (equal); Validation (lead); Visualization (equal); Writing‐original draft (lead); Writing‐review & editing (lead). **Joseph Petrosino:** Conceptualization (supporting); Formal analysis (supporting); Funding acquisition (lead); Resources (lead); Visualization (equal); Writing‐review & editing (supporting).

## Data Availability

The data generated or analyzed during this study are included in this published article. Raw source data for figures are publicly available on figshare (Figure 1: https://doi.org/10.6084/m9.figshare.13604402; Figure 2: https://doi.org/10.6084/m9.figshare.13604405; Figure 3: https://doi.org/10.6084/m9.figshare.13604411; Figure 4: https://doi.org/10.6084/m9.figshare.13604417).

## APPENDIX A

**TABLE A1:** Primers used in this study

Name	Sequence (5′ → 3′)
*Retargeting TargeTron^®^ template DNA*	
EBS Universal (Sigma)	CGAAATTAGAAACTTGCGTTCAGTAAC
*clcA*	
0095‐534‐IBS	AAAACTCGAGATAATTATCCTTAGGACTCGCAGCTGTGCGCCCAGATAG
0095‐534‐EBS1d	CAGATTGTACAAATGTGGTGATAACAGATAAGTCGCAGCTGCTAACTTACCTTTCTTTGT
0095‐534‐EBS2	TGAACGCAAGTTTCTAATTTCGATTAGTCCTCGATAGAGGAAAGTGTCT
*Plasmid verification and sequencing*	
TT Forward	GCAAGAGCTTGGAACTTTGAG
TT Reverse	GCATTCTTGTTTAGGGTATCCC
*TargeTron insertion verification*	
0095 Forward	CGACATATTTGGGTGCTAGTAC
0095 Reverse	GCAATCAAAAGCCAACTCCAAA
*Kan Probe (Rodriguez et al., 2009)*	
Forward	TGCATGGTTACTCACCACTGC
Reverse	TACAACCTATTAATTTCCCCTCG
*Intron Specific (Rodriguez et al., 2009)*	
Forward	TAGGAGAACCTATGGGAACGAAACG
Reverse	ACTGTACCCCTTTGCCATGT
*Quantitative Real Time RT‐PCR*	
Murine β‐actin Forward	CAGCCTTCCTTCTTGGGTATG
Murine β‐actin Reverse	TCTTCATGGTGCTAGGAGCC
Murine IL‐18 Forward	TCTTGGCCCAGGAACAATGG
Murine IL‐18 Reverse	GGTTGTACAGTGAAGTCGGC
Murine IL‐6 Forward	CCAAGAGATAAGCTGGAGTCACA
Murine IL‐6 Reverse	AACGCACTAGGTTTGCCGAG
Murine IL‐12 Forward	TCTGCTGCTCCACAAGAAGG
Murine IL‐12 Reverse	AGGGGAACTGCTACTGCTCT
Murine IL‐1β Forward	GCCACCTTTTGACAGTGATGAG
Murine IL‐1β Reverse	GACAGCCCAGGTCAAAGGTT
Murine TNF‐α Forward	CACAGAAAGCATGATCCGCGACGT
Murine TNF‐α Reverse	CGGCAGAGAGGAGGTTGACTTTCT
Human β‐actin Forward	CACAGAGCCTCGCCTTTGC
Human β‐actin Reverse	ATATCATCATCCATGGTGAGCTGG
Human IL‐18 Forward	AAACCTGGAATCAGATTACTTTGG
Human IL‐18 Reverse	TCCGGGGTGCATTATCTCTAC
Human IL‐1β Forward	GCCAATCTTCATTGCTCAAGTGT
Human IL‐1β Reverse	GGTCGGAGATTCGTAGCTGG
Human TNF‐α Forward	TCAGCTTGAGGGTTTGCTAC
Human TNF‐α Reverse	TGCACTTTGGAGTGATCGG

**TABLE A2:** TargeTron^®^ plasmids retargeted for *Francisella* candidate attenuation genes

Name	Description	References
pKEK1140	*Francisella tularensis* TargeTron^®^ vector, blue‐white screening	Klose (1, 2)
pKEK1137	pKEK1140 retargeted for FTH1789	Klose (1, 2)
pJFP1001	pKEK1140 retargeted for FTH1558	This study^a^
pJFP1002	pKEK1140 retargeted for FTH0800	This study^b^
pJFP1003	pKEK1140 retargeted for FTH0836	This study^a^
pJFP1004	pKEK1140 retargeted for FTH0095 (*clcA)*	This study^a^
pJFP1005	pKEK1140 retargeted for FTH0374	This study^b^
pJFP1006	pKEK1140 retargeted for FTH0384	This study^a^
pJFP1007	pKEK1140 retargeted for FTH0431	This study^a^
pJFP1008	pKEK1140 retargeted for FTH0207	This study^b^
pJFP1009	pKEK1140 retargeted for FTH0534	This study^b^
pJFP1010	pKEK1140 retargeted for FTH1503	This study^b^
pJFP1011	pKEK1140 retargeted for FTH0432	This study^b^
pJFP1012	pKEK1140 retargeted for FTH1042	This study^b^
pJFP1013	pKEK1140 retargeted for FTH1116	This study^b^
pJFP1014	pKEK1140 retargeted for FTH1709	This study^b^
pJFP1015	pKEK1140 retargeted for FTH1471	This study^a^
pJFP1016	pKEK1140 retargeted for FTH1511	This study^b^
pJFP1017	pKEK1140 retargeted for FTH1471	This study^a^
pJFP1018	pKEK1140 retargeted for *mglA* (FTH1275)	This study^a^

Note

All constructs sequence‐verified by L. Matz.

^a^ Engineered by L. Matz.

^b^ Engineered by S. Herb with help from T. Ayvaz.
